# COVID-19-associated MRSA infective endocarditis and mitral valve perforation: a case report

**DOI:** 10.1186/s43044-023-00392-z

**Published:** 2023-07-19

**Authors:** Anwar Khedr, Esraa Mamdouh Hassan, Hussam Al Hennawi, Abbas Bashir Jama, Muhammad Khuzzaim Khan, Mikael Mir, Aalaa Eissa, Ibtisam Rauf, Hisham Mushtaq, Nitesh Kumar Jain, Mir Rauf Subla, Salim Surani, Syed Anjum Khan

**Affiliations:** 1 BronxCare Health System, Bronx, NY USA; 2 Mayo Clinic Health System, Mankato, MN USA; 3 Jefferson Health System, Abington, PA USA; 4 Dow University of Health Sciences, Mission Rd, New Labour Colony Nanakwara, Karachi, 74200 Pakistan; 5 University of Minnesota Medical School, Minneapolis, MN USA; 6 Kafrelsheikh University Hospital, Kafr el-Sheikh, Egypt; 7 St. George’s School of Medicine, University Centre Grenada, West Indies, Grenada; 8 Texas A&M University, College Station, TX USA

**Keywords:** MRSA, COVID-19, Staphylococcus aureus, Endocarditis, Mitral perforation

## Abstract

**Background:**

Coronavirus disease 2019 (COVID-19) has emerged as a global pandemic, leading to significant morbidity and mortality. The interplay between COVID-19 and other medical conditions can complicate diagnosis and management, necessitating further exploration.

**Case presentation:**

This case report presents a patient with COVID-19 who developed infective endocarditis (IE) and mitral valve perforation caused by methicillin-resistant Staphylococcus aureus on a native mitral valve. Notably, the patient did not exhibit typical IE risk factors, such as intravenous drug use. However, he did possess risk factors for bacteremia, including a history of diabetes mellitus and recent steroid use due to the COVID-19 infection. The diagnosis of IE was crucially facilitated by transesophageal echocardiography.

**Conclusions:**

This case highlights the potential association between COVID-19 and the development of infective endocarditis. Prompt evaluation using transesophageal echocardiography is vital when there is a high suspicion of IE in COVID-19 patients. Further research is required to elucidate the precise relationship between COVID-19 and IE.

## Background

Infective endocarditis (IE) is a serious bacterial infection that occurs when bacteria enter the bloodstream and form vegetations on the heart lining, heart valve, or blood vessel. The most common causative agent is Staphylococcus aureus, which can lead to complications such as emboli and abscesses in distant organs such as the central nervous system, spleen, kidneys, or skin. IE has a high mortality and morbidity rate due to the risk of embolic phenomena [1, 2].

The emergence of coronavirus disease 2019 (COVID-19) presents new challenges for diagnosing bacterial IE, as both diseases can present with similar symptoms such as fever, chills, dyspnea, fatigue, cough, and myalgia [3]. Transesophageal echocardiography (TEE) may be required for early detection and treatment of suspected IE in individuals with severe COVID-19 pneumonia who are admitted to intensive care units [1]. However, performing TEE was difficult in the early stages of the pandemic due to concerns about infection risk during the procedure [4].

Preexisting cardiovascular diseases are a significant risk factor for higher morbidity and mortality in patients with severe acute respiratory syndrome coronavirus 2 (SARS-CoV-2). However, compared to other cardiac diseases, IE is less frequently reported among patients infected with COVID-19 [5]. In this case report, we present a patient with COVID-19 who developed IE and mitral valve perforation caused by Staphylococcus aureus on a structurally normal native mitral valve. Despite the absence of traditional risk factors for IE, the patient had a history of diabetes mellitus and recent steroid treatment, which increased the risk of bacteremia.

## Case presentation

A 67-year-old male patient was referred to our medical center for further management of COVID-19 associated pneumonia. The patient had a history of hypertension, hyperlipidemia, obesity, insulin-dependent diabetes mellitus, coronary artery disease, recurrent bleeding duodenal ulcer, and obstructive sleep apnea. He presented to an outside hospital with mild symptoms of COVID-19 and was considered for monoclonal antibody infusion treatment. He had not received COVID-19 vaccination at that time. Four days later, the patient became febrile and hypoxic, requiring hospitalization.

**Fig. 1:**
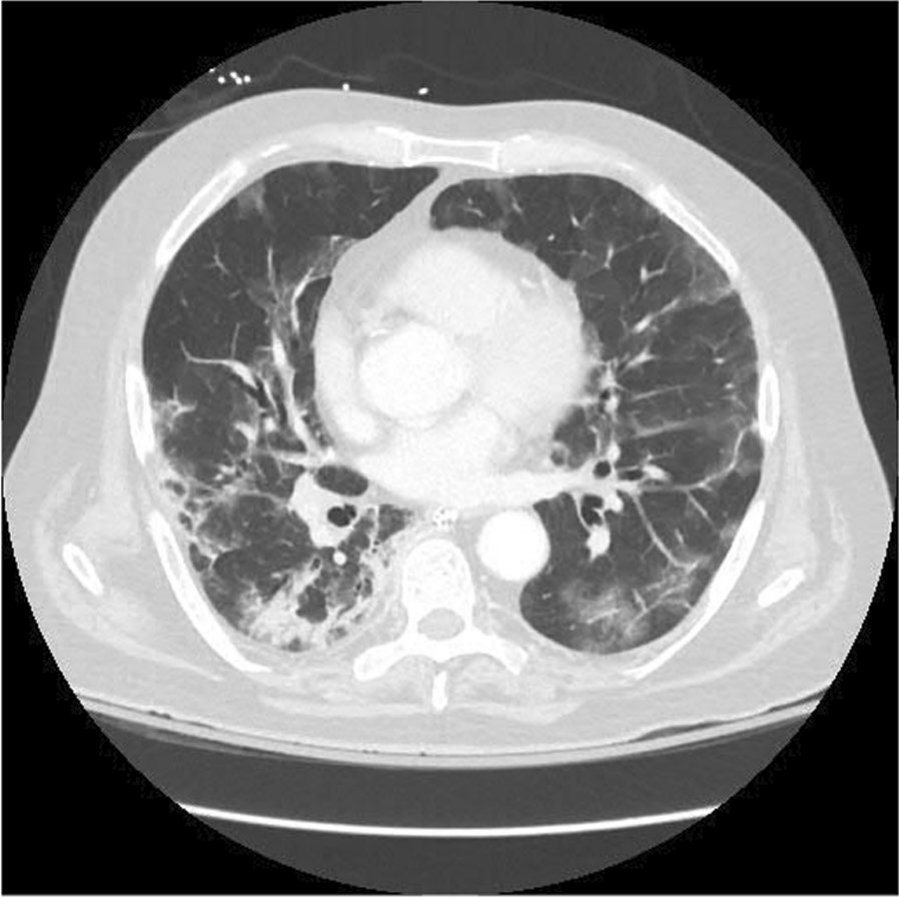
Computed tomography of the chest showing progressive COVID-19 pneumonia with bilateral ground-glass opacities and consolidation

Upon admission, the patient was alert and oriented with a blood pressure of 106/73 mmHg, pulse rate of 125 beats per minute, body temperature of 38.5 °C, and respiratory rate of 35 breaths per minute. The oxygen saturation was 89% on room air and 95% with the use of a high flow nasal cannula. A physical examination revealed diminished breath sounds in all lung fields. Laboratory tests were ordered, with the abnormal results shown in Table 1. An ultrasound of the lower extremities showed an acute left deep vein thrombosis (DVT) extending to the common femoral vein. A chest CT scan showed progressive COVID-19 pneumonia and was negative for pulmonary embolism (Fig. 1).

**Table 1:** Abnormal lab results on hospital admission

Laboratory tests	On admission	Reference range
*CBC without differential*		
Hemoglobin (g/dL)	10.4	13.2–16.6
Hematocrit (%)	31.5	38.3–48.6
Erythrocytes (× 10^12^/L)	3.74	4.35–5.65
RBC distribution width (%)	14.8	11.8–14.5
Platelet count (× 10^9^/L)	330	135–317
Leukocytes (× 10^9^/L)	10.4	3.4–9.6
*Coagulation profile*		
Activated partial thromboplastin time (APTT) (s)	56	25–37
*Hepatic function panel*		
Aspartate aminotransferase (AST) (U/L)	54	8–48
Albumin (g/dL)	2.8	3.5–5.0
Protein, total (g/dL)	5.6	6.3–7.9
*Basic metabolic panel*		
Sodium (mmol/L)	129	135–145
Calcium, total (mg/dL)	8.1	8.8–10.2
Glucose (mg/dL)	169	70–140
Anion gap	6	7–15
Creatinine (mg/dL)	0.63	0.74–1.35

**Fig. 2:**
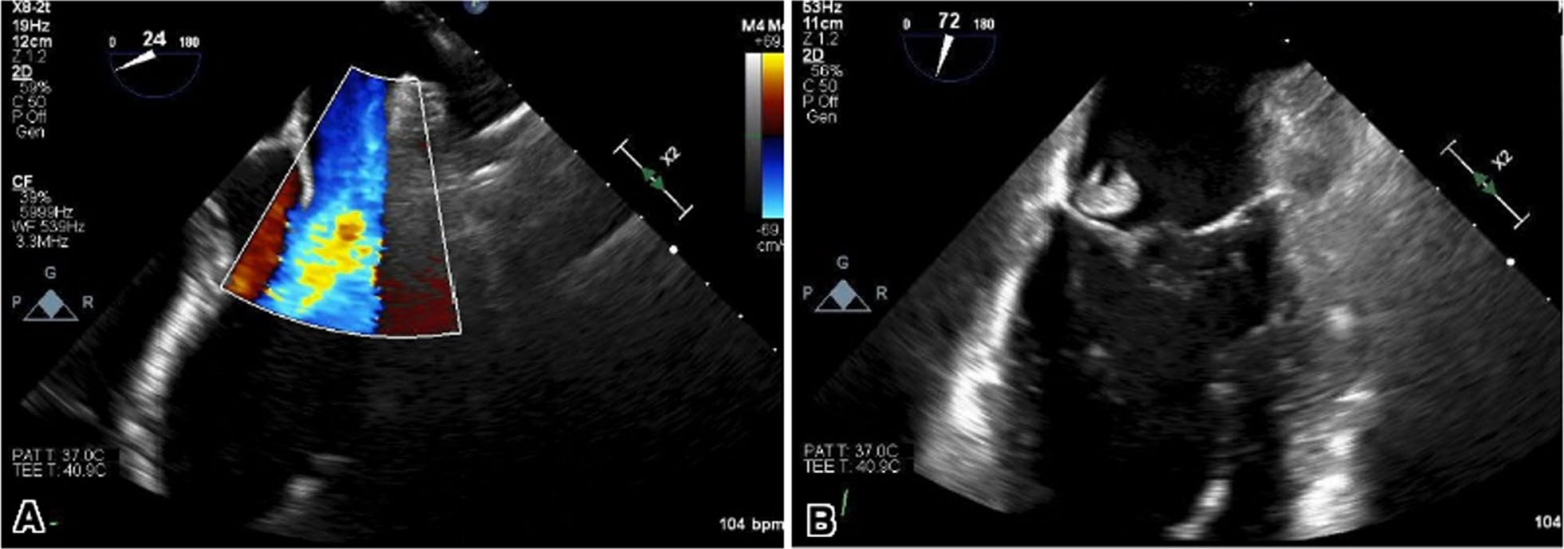
(**A**) Transesophageal echocardiography demonstrating mitral regurgitation depicted by moderate to a severe posterior eccentric jet of mitral regurgitation with evidence of leaflet perforation and color Doppler signal going through the vegetation. (**B**) Large and rounded echo density measuring up to 1.2 × 1.3 cm attached to the P3 segment of the posterior mitral valve leaflet suggestive of endocarditis, mitral vegetation, and perforation

The patient was treated with heparin, remdesivir, dexamethasone, and baricitinib for one week, but he continued to clinically deteriorate with increased work of breathing, progressive hypoxemia, and high-grade fever, necessitating a transfer to ICU, where he was intubated and received mechanical ventilation. Further investigations were conducted, including a repeat ultrasound of the lower extremities, which showed the thrombus no longer extended to the common femoral vein. His lactate was noted to be elevated, and blood cultures were obtained, which were positive for methicillin-resistant Staphylococcus aureus (MRSA) and the patient was started on IV Vancomycin. The transesophageal echocardiogram (TEE) revealed evidence of native mitral valve endocarditis, characterized by moderate to severe regurgitation and a potential perforation (Fig. 2A, B). Importantly, the TEE did not indicate the presence of a patent foramen ovale.

The patient was transferred to the cardiac ICU and continued on IV Vancomycin. Repeat TEE showed a persistent mobile echo density consistent with vegetation adherent to the atrial aspect of the mitral P3, with an endocarditis-related perforation of the posterior mitral valve and moderate regurgitation. No evidence of perivalvular extension or endocarditis was found on the remaining cardiac valves, and the mitral vegetation appeared slightly smaller than in previous imaging. To assess the electrical activity of the heart, an electrocardiogram (ECG) was performed, yielding normal results without any indications of heart block. The patient was treated with IV Lasix, Losartan, and Metoprolol for acute severe mitral regurgitation, aimed at reducing preload and afterload.

A brain MRI was performed in the setting of endocarditis and the possible need for systemic anticoagulation but was negative for any acute pathology, including stroke or emboli. The patient was discharged on IV vancomycin for six weeks and was not deemed a candidate for emergency surgery as he was hemodynamically stable. However, one week later, the patient developed a peripherally inserted central catheter (PICC) thrombus extending into the right ventricular outflow tract. He underwent a successful percutaneous evacuation of the thrombus. Four days later, he also underwent a successful mitral valve replacement.

## Discussion

The COVID-19 pandemic has had a significant impact on the economy, politics, and health globally, and it was declared a pandemic by the World Health Organization [6]. Most patients experience fever and cough as the predominant symptoms, which in severe cases, can lead to acute respiratory distress syndrome, multi-organ failure, and death [7]. The literature suggests that preexisting cardiac disease can increase the risk of morbidity and mortality in COVID-19 patients. The evidence regarding the incidence of IE in the setting of COVID-19 is scarce. There have been some case reports which discussed the association of COVID-19 with the development of IE, however, it remains unclear whether both diseases interact and impact each other's course of treatment and management [6].

It is important to recognize that severe COVID-19 cases can result in substantial long-term consequences [8]. Our patient had COVID-19, and was treated with steroids, which led to decreased immunity and bacteremia, and it is possible that it contributed to the development of IE. The suspicion was high as he met one major Duke criterion (TEE revealing evidence of native mitral valve endocarditis with moderate to severe regurgitation and possible concomitant perforation) and three minor criteria (fever, the vascular phenomenon of DVT, and blood culture positive for MRSA) [8]. The hypercoagulable condition created by the recent COVID-19 infection may have played a role as a risk factor in causing damage to the mitral valve structure, which could have contributed to the development of IE. This might also be incorrect as the process of vegetation starts through transient bacteremia, followed by binding and adherence to the damaged endothelium and encasing in a platelet/fibrin matrix [9]. The damage to the mitral valve structure could also have been caused by the recent COVID-19 infection [10]. However, it is also possible that the source of bacteremia was positive sputum culture for MRSA, leading to the development of IE.

During the COVID-19 pandemic, physicians have faced the challenge of determining the role of COVID-19 in a patient’s presentation of other disorders [11]. This may be especially difficult in patients with acute IE due to similar symptoms, such as shortness of breath, fatigue, and fever, which are also common in COVID-19 infection [11]. A rapid and accurate diagnosis with TEE is critical in the case of acute IE, as delayed treatment may result in death [11]. Our patient's CT scan was more consistent with COVID-19 pneumonia than congestive heart failure due to mitral valve dysfunction. As demonstrated in our case, TEE is an important tool in the diagnosis of IE. Urgent and long-term antibiotic therapy is crucial for treating the infection, and surgical intervention may also be required if the valve is destroyed [11]. Current recommendations indicate that once a surgical indication has been established, the procedure should be carried out as soon as possible [12].

A recent systematic review reported 21 cases of patients with previous COVID-19 infection who developed IE, with only two of them being caused by MRSA [13]. Another systematic review reported 15 patients with active COVID-19 infection and a concurrent development of IE, with three patients having MRSA as the causative agent. Both the aortic and mitral valves were found to be equally at risk of infection [14].

## Conclusions

The COVID-19 pandemic has posed challenges for the diagnosis of IE, particularly in patients who are also infected with COVID-19. In these cases, it is crucial for clinicians to perform a comprehensive cardiac examination with TEE to diagnose IE as early as possible. This case highlights the potential severity of the consequences of COVID-19 and the difficulties in diagnosing and managing co-existing diseases, especially in immunocompromised patients. Further research is needed to establish a direct relationship between COVID-19 and IE, but this case serves as a reminder to physicians to remain vigilant in their approach to similar cases in the future.

## Data Availability

Not applicable.

## Abbreviations

IE: Infective endocarditis
COVID-19: Coronavirus disease 2019
MRI: Magnetic resonance imaging
TEE: Transesophageal echocardiography
SARS-CoV-2: Severe acute respiratory syndrome coronavirus 2
MRSA: Methicillin-resistant Staphylococcus aureus
PICC: Peripherally inserted central catheter
DVT: Deep vein thrombosis
ECG: Electro-cardiogram
